# Conserved Pattern and Potential Role of Recurrent Deletions in SARS-CoV-2 Evolution

**DOI:** 10.1128/spectrum.02191-21

**Published:** 2022-03-07

**Authors:** Shenghui Weng, Hangyu Zhou, Chengyang Ji, Liang Li, Na Han, Rong Yang, Jingzhe Shang, Aiping Wu

**Affiliations:** a Institute of Systems Medicine, Chinese Academy of Medical Sciences & Peking Union Medical College, Beijing, China; b Suzhou Institute of Systems Medicine, Suzhou, China; c Linyi People’s Hospital, Shandong, China; Erasmus MC

**Keywords:** SARS-CoV-2, recurrent deletion, mutation, structural variation, adaptive evolution

## Abstract

SARS-CoV-2 continues adapting to human hosts during the current worldwide pandemic since 2019. This virus evolves through multiple means, such as single nucleotide mutations and structural variations, which has brought great difficulty to disease prevention and control of COVID-19. Structural variation, including multiple nucleotide changes like insertions and deletions, has a greater impact relative to single nucleotide mutation on both genome structures and protein functions. In this study, we found that deletion occurred frequently in not only SARS-CoV-2 but also in other SARS-related coronaviruses. These deletions showed obvious location bias and formed 45 recurrent deletion regions in the viral genome. Some of these deletions showed proliferation advantages, including four high-frequency deletions (nsp6 Δ106-109, S Δ69-70, S Δ144, and Δ28271) that were detected in around 50% of SARS-CoV-2 genomes and other 19 median-frequency deletions. In addition, the association between deletions and the WHO reported variants of concern (VOC) and variants of interest (VOI) of SARS-CoV-2 indicated that these variants had a unique combination of deletion patterns. In the spike (S) protein, the deletions in SARS-CoV-2 were mainly in the N-terminal domain. Some deletions, such as S Δ144/145 and S Δ243-244, have been confirmed to block the binding sites of neutralizing antibodies. Overall, this study revealed a conservative regional pattern and the potential effect of some deletions in SARS-CoV-2 over the whole genome, providing important evidence for potential epidemic control and vaccine development.

**IMPORTANCE** Mutations in SARS-CoV-2 were studied extensively, while only the structure variations on the spike protein were discussed well in previous studies. To study the role of structural variations in virus evolution, we described the distribution of structure variations on the whole genome. Conserved patterns were found of deletions among SARS-CoV-2, SARS-CoV-2-like, and SARS-CoV-like viruses. There were 45 recurrent deletion regions (RDRs) in SARS-CoV-2 generated through the integration of deleted positions. In these regions, four high-frequency deletions parallelly appeared in multiple strains. Furthermore, in the spike protein, the deletions in SARS-CoV-2 were mainly in the N-terminal domain, blocking the binding sites of some neutralizing antibodies, while the structural variations in SARS-related coronavirus were mainly in the N-terminal domain and receptor binding domain. The receptor binding domain is highly related to hosting recognition. The deletions in the receptor binding domain may play a role in host adaption.

Since the outbreak of COVID-19 caused by SARS-CoV-2 in late 2019, this virus has spread globally for nearly two years. In some regions, although the vaccination rate or infection rate has reached a relatively high level, great concerns have been raised for the continuous variation in SARS-CoV-2 on their ability to escape immune neutralization (1, 2). Recently, a SARS-CoV-2 variant, B.1.617.2, also named the Delta strain by World Health Organization (WHO), has shown increased transmission and immune escape capabilities (3, 4). People with previously induced antibodies still have the risk of infection by this variant (5). Therefore, there is an urgent need to understand the molecular mechanism underlying the adaptive evolution of SARS-CoV-2.

SARS-CoV-2 can take advantage of genome variation to evolve rapidly, including single nucleotide polymorphisms (SNPs) and structural variations (SVs). SVs consist of short fragment insertions, deletions, sequence reversals, and recombination, etc. Current research mainly focused on SNPs (6), but SV changes can include more nucleotides, which may have a greater impact on genomic structure or protein function. Many SVs arise during a viral passage, while only a small part can be retained and spread. These preserved deletions may have played a potential role during the evolution of SARS-CoV-2 (7).

Previous studies have shown that fragment deletions have the possibility to affect the proliferation and transmission of SARS-CoV-2 (8, 9). For instance, a 382-nucleotide deletion in the ORF8 protein weakening the virulence of SARS-CoV-2 was reported in the early stages of the SARS-CoV-2 epidemic in Singapore (8). A Δ500-532 deletion event was shown to reduce the host INF-β response, a mutation that seemed to occur early in this epidemic and can be found on the nonstructural protein 1 (nsp1) (10). Another 34-nucleotide deletion was found in France on the ORF6 protein. This variant was shown to induce the overexpression of several specific cytokines, including CCL2/MCP1, PTX3, and TNFα, etc., which are involved in the regulation and transduction of NF-kb signaling (11). Recently, in the B.1.1.7 lineage of SARS-CoV-2, Δ69-70 and Δ144 were found in the S protein. S Δ69-70 was shown to increase the viruses’ ability to release the S2 structure, which can augment viral infectivity and improve viral syncytium production (12). Based on a bioinformatic analysis, Reham et al. found S Δ144 can alter the pocket structure on the N-terminal (NTD) of the S protein and reduce the affinity between the NTD and endogenous host antibodies (13).

With the accumulation of site information and structural variations, more SARS-CoV-2 variants with divergent mutations continue to appear in the literature. For instance, the B.1.1.7 variant (the Alpha strain) outbreak occurred in the United Kingdom first, then the B.1.617.2 variant (the Delta strain) outbreak happened in India. These variants have been observed to evade vaccine immunity (3, 14). Four recurrent deletion regions (RDRs), including S Δ69-70 and S Δ144, in the S protein, prevent the virus from being bound by neutralizing antibodies (15). The above observations suggest that deletion is one of the ways for SARS-CoV-2 to escape from adaptive immunity and to adapt to their host. Therefore, a systematical analysis of the pattern of deletions in SARS-CoV-2 and their potential effect on immune escape is urgently needed.

In this study, we comprehensively analyzed deletions and insertions in SARS-CoV-2, together with those in SARS-CoV-2-like and SARS-CoV-like viruses. We found that there were conserved patterns of deletions not only in SARS-CoV-2 but also in SARS-CoV-2-like and SARS-CoV-like viruses. Among all recurrent deletion regions (RDRs), SARS-CoV-2 evolved four high-frequency deletions that were found in over 48% of sequenced strains, which were mostly the dominant Alpha strain (lineage B.1.1.7). It is worth noting that the deletions from RDRs were detected in all six variants of concern as defined by the WHO (16) with different combinations. Furthermore, the NTD and RBD regions of the S protein possess multiple RDR regions, which may promote rapid viral adaptation to the host.

## RESULTS

### Common RDRs in SARS-CoV-2, SARS-CoV-2-like, and SARS-CoV-like viruses.

**FIG 1:**
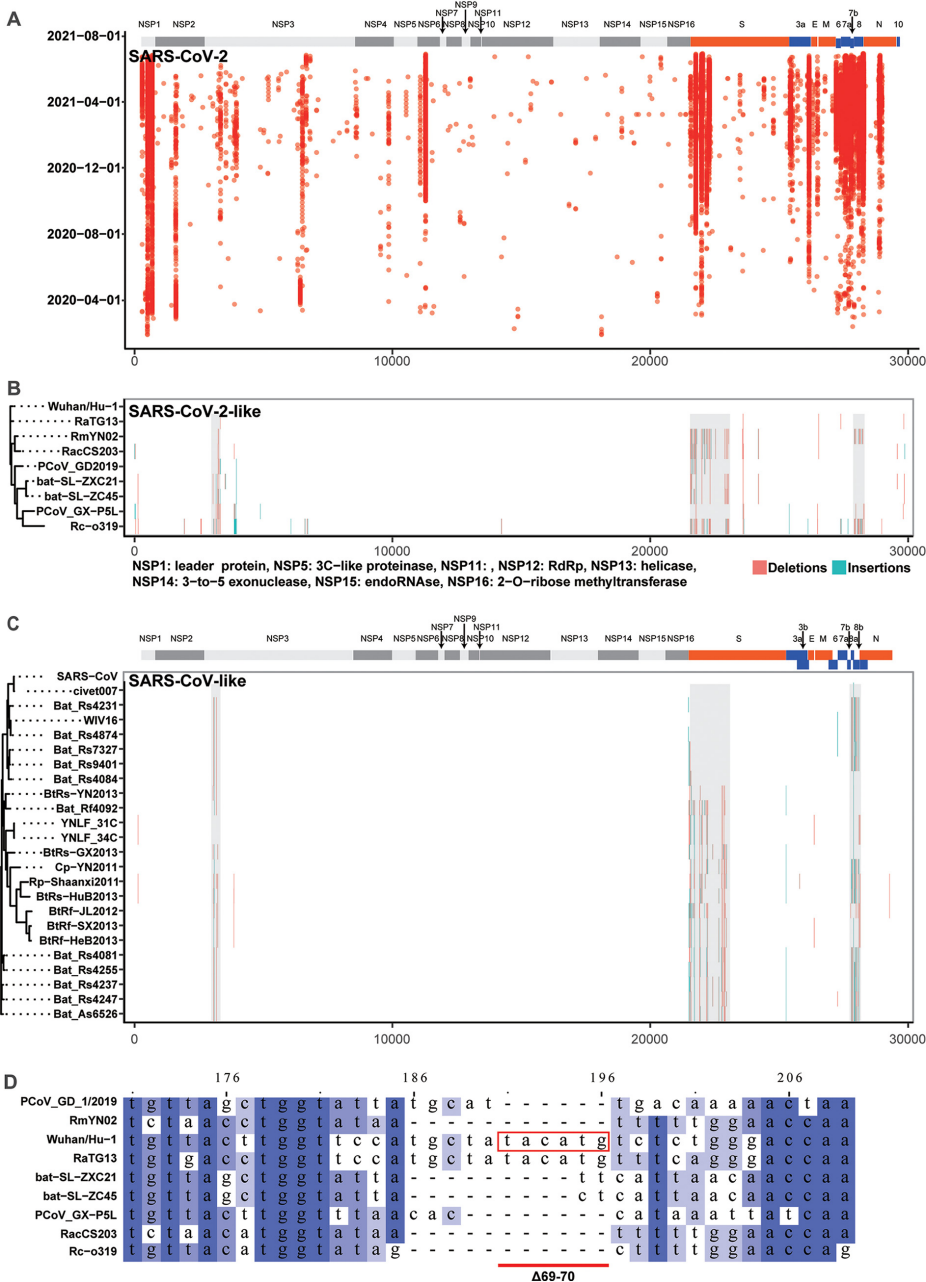
The genomic distribution of insertions and deletions in SARS-CoV-2 and SARS-CoV-2-like viruses. (A) Deletions are dotted in the SARS-CoV-2 genome (X-axis) with time (Y-axis). The distribution of insertions (blue) and deletions (red) in SARS-CoV-2-like (B) and SARS-CoV-like viruses (C) are shown in the same way. Three shared high deletion/insertion areas 1-3 (HDAs 1-3) are highlighted with grey boxes. The reference genomes are EPI_ISL_402124 and NC_004718.3 for SARS-CoV-2 and SARS-CoV, respectively. (D) Context of S Δ69-70 in aligned SARS-CoV-2-like virus genome sequences. S Δ69-70 of SARS-CoV-2-like viruses is framed out by a red rectangle.

Previous studies have shown a regional preference of deletions in the NTD domain of the S protein in SARS-CoV-2, forming four recurrent deletion regions (RDR1-4) (15). Here, we systematically analyzed these deletion and insertion events in 1,289,583 high-quality SARS-CoV-2 genomes downloaded on July 05, 2021, from GISAID (see in Method). In total, 1007 unique deletions and 387 unique insertions (Table S1 and S2) were detected. The maximum number of occurrences of these unique insertions was only 397 times in the dataset, while that of these unique deletions was 685,744 (53.18%) times, which indicates that deletions were the major structural variations in SARS-CoV-2. Across the entire genome, these deletions showed a clear regional preference (Fig. 1A), which mainly occurred in the nsp1, nsp2, nsp3, nsp4, nsp6, S, N proteins, and accessory proteins (Fig. S1A). After the integration of these deleted positions, 45 RDRs were generated (Table S3). Furthermore, we found that the diversity of deletions increased with time significantly (Fig. S1B).

To investigate these biased RDRs in other coronaviruses, we collected genome sequences for SARS-CoV-2-like and SARS-CoV-like viruses from different hosts and generated a set of deletions referring to SARS-CoV-2 or SARS-CoV (Table S4). In these sequences, we found that most deletions were in three regions, forming three high deletion/insertion areas (HDA 1-3) in the front part of the nsp3, S, and ORF8, respectively (Fig. 1B and C). The pattern indicated that these three HDAs were conserved among SARS-related coronaviruses. In these three proteins in the SARS-CoV-2 genome, there were twelve RDRs, seven RDRs, and one 436-nt large RDR, respectively. These facts led us to speculate the roles of these RDRs and HDAs in the evolution of coronaviruses. In addition, when we pulled out the aligned sequences of S Δ69-70 in SARS-CoV-2-like viruses, we found that the high-frequency S Δ69-70 deletion in SARS-CoV-2 also existed in these SARS-related sequences (Fig. 1D). This finding further indicated that deletions in HDAs were not randomly distributed. These deletions with a location preference could be the result of adaptive selection.

### Diversity of deletion types in RDRs.

**FIG 2:**
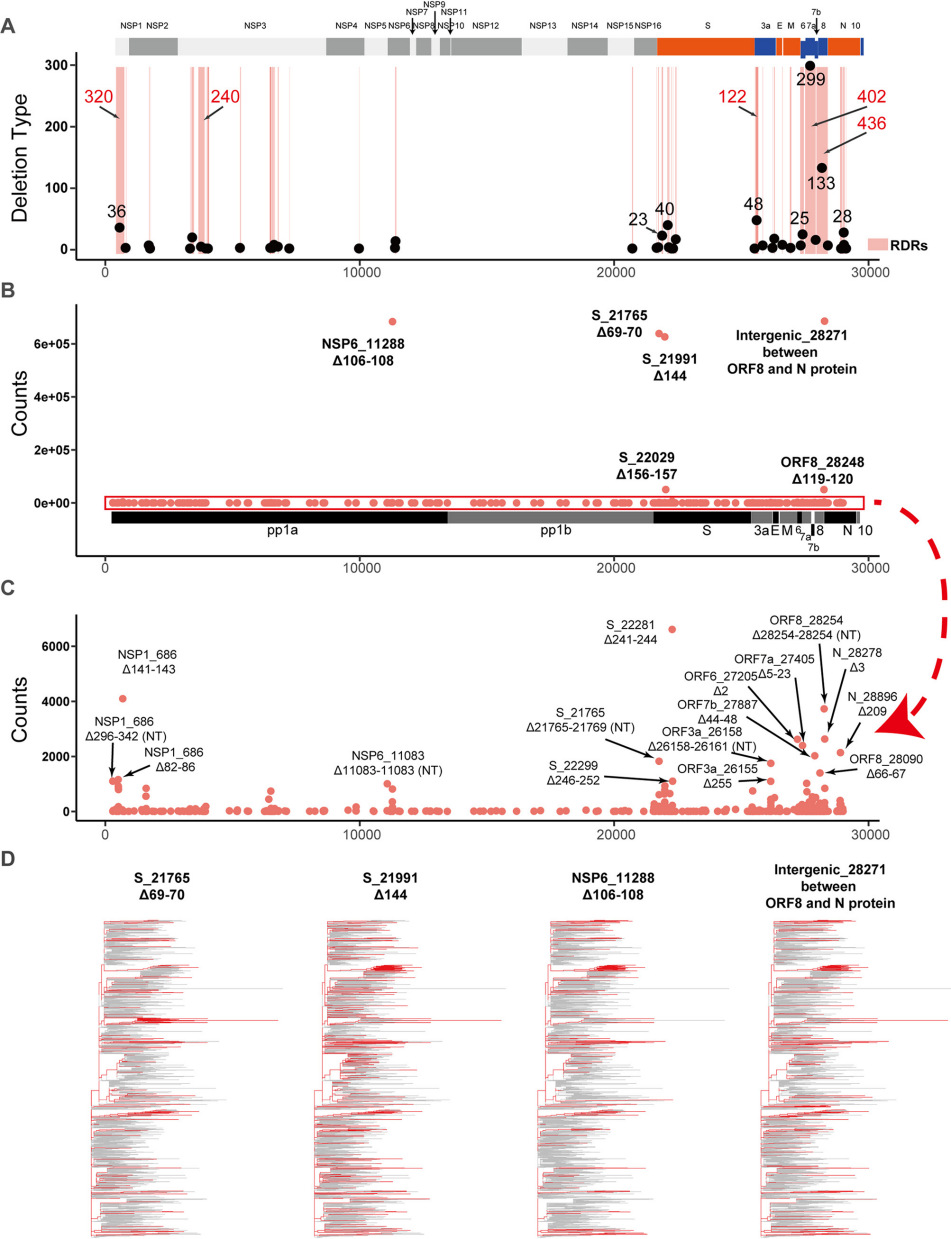
Deletion types in the RDR regions of the SARS-CoV-2 genome. (A) The distribution of RDR regions on the SARS-CoV-2 genome together with the number of deletion types in each RDR. The RDR regions are in red boxes. The length of the regions that are longer than 100 nt is labeled under their boxes. The black points on the red boxes represent the count of their pattern type. The counts larger than 20 times are labeled out above the points. The high-frequency (B) and medium-frequency (C) deletions were observed in the SARS-CoV-2 genome. High-frequency deletions occurred more than 600, 000 times, and middle-frequency deletions showed more than 1, 000 times. (D) The variants containing four high-frequency deletions (red) are highlighted in the phylogenetic tree of SARS-CoV-2. The other variants are colored in grey as background.

Among the 45 RDRs in the SARS-CoV-2 genome, the distribution of the deletions in RDRs showed location-dependent characteristics. In the S protein, RDRs were located in its NTD domain. In the first fifth of the ORF3a sequence, there was one long 122-nt RDR. RDRs were identified out in a cluster between the M protein and N protein, also covering four accessory proteins (ORF6, ORF7Aa, ORF7b, and ORF8). Two longest RDRs were involved in this cluster, including one 402-nt RDR and one 436-nt RDR (Fig. 2A). The discontinuous transcription mechanism of SARS-CoV-2 may be the reason underlying the location preference of these RDRs (17). When we studied the association between the length and the deletion type of these RDRs, we found that more deletion types were identified in longer RDRs. In the five longest RDRs, there were more than 30 deletion types detected. Especially, among the 240-nt RDR in the nsp3, 5 deletion types have been identified. While in the S protein, among the 49-nt RDR22 and 37-nt RDR23, their deletion types were as high as 32 and 42, respectively. The relatively high diversity of these short RDRs of S protein could be due to the important role of the S protein in the adaptive evolution of the SARS-CoV-2 virus. These results showed that deletions are prone to occur in some specific RDRs.

Though there were 45 RDRs, including 842 deletion types that have been identified (Table S3) in the SARS-CoV-2 genome, only 4 high-frequency deletions were observed in over 600,000 strains (48.58%) among all genomes (Fig. 2B). They were nsp6 Δ106-108, Δ28271 (a single-nucleotide intergenic deletion before the protein N), and two widely studied deletions, S Δ69-70 and S Δ144. It is worth noting that two of these deletions, S Δ69-70 and S Δ144, have been reported to occur spontaneously in immunodeficient patients (12, 18). In addition, 19 median-frequency deletions were identified in the nsp1, nsp6, S, and N, as well as four accessory proteins (ORF3a, ORF6, ORF7a, and ORF8) (Fig. 2C). By analyzing the temporal and spatial distribution of these four high-frequency deletions, we found that these deletions shared a similar growth pattern on six continents. They all emerged first in Europe at the end of 2020 and then spread to other regions (Fig. S2). Phylogenetic trees were used to analyze whether these mutations were distributed repeatedly and widely. The results showed that these four high-frequency deletions were distributed in parallel branches (Fig. 2D). Their repeated and independent occurrence pattern indicated their potential advantages for viral adaption.

### Relationships between deletions and SARS-CoV-2 variants.

**FIG 3:**
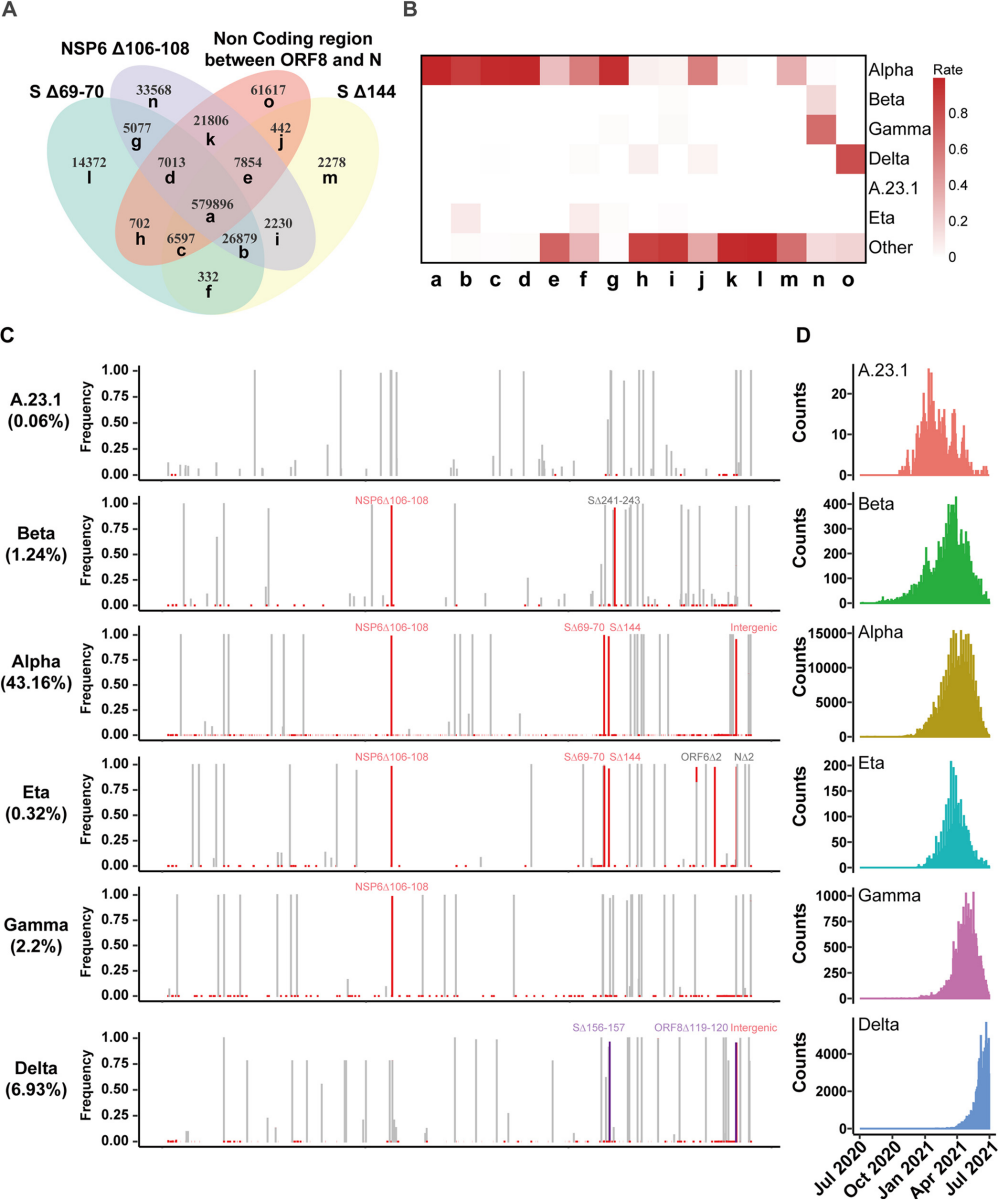
Deletions in different SARS-CoV-2 variants. (A) The number of overlapping sequences of high-frequency deletions in SARS-CoV-2 is shown in a Venn diagram. Group a-o represents each part in the Venn diagram. (B) Group a-o consists of various forms of deletion combination. The gradation of color represents the rate of each combination in each strain. The sum of each column is 1. (C) Deletions (red) and mutations (grey) are mapped in six VOC and VOI SARS-CoV-2 strains. The high-frequency and median-frequency deletions are colored in red and purple, respectively. (D) The number of daily samples of VOC and VOI strains is displayed in bar plots.

Four high-frequency deletions were found in a similar spatiotemporal pattern, which indicated there were internal associations between them. Venn diagram analysis showed that most of these four deletions appeared together in about 580,000 (45%) sequenced SARS-CoV-2 genomes (Fig. 3A). Except for the cooccurrence of four high-frequency deletions, other combinations among these four deletions also occurred repeatedly. We named each combination of deletions from group a to group o (Fig. 3B). Five of these combinations were observed more than 10,000 times, which were relatively frequent since this was higher than that of the middle-frequency deletions shown in Fig. 2C. Recently, the WHO reported variants of concern (VOC) and variants of interest (VOI) of SARS-CoV-2 based on their potential transmission risk and immune escape abilities. When we calculated the ratios of each combination in different variants, we found that except for the combination of four high-frequency deletions, the combination with three of four high-frequency deletions were also mainly belonged to the Alpha strain (lineage B.1.1.7). Group n with only nsp6 deletion belonged to the Beta (lineage B.1.351) and the Gamma strains (lineage P.1). Group o with the intergenic deletion mainly belonged to the Delta strain (lineage B.1.617.2). Other groups (h, I, k, and l) widely distributed in different SARS-CoV-2 lineages (Fig. 3B).

Apart from four high-frequency deletions, we observed that each SARS-CoV-2 variant had its unique combination of deletions and mutations (Fig. 3C). The Alpha variant (lineage B.1.1.7) contained all four high-frequency deletions, while other variants contained only a part of these deletions. In addition, we found that the deletion Δ241-243 in the S protein appeared in the Beta variant (lineage B.1.351), while the deletions ORF6 Δ2 and N Δ2 appeared in the Eta variant (lineage B.1.525). Notably, the recent SARS-CoV-2 variant Delta (lineage B.1.617.2), contained two intermediate-frequency deletions, namely, S Δ156-157 and ORF8 Δ119-120 (Fig. 3D). These results showed that variants formed at different times and in different environments carried their unique deletions and mutations.

### Association of deletions and mutations in the S protein.

**FIG 4:**
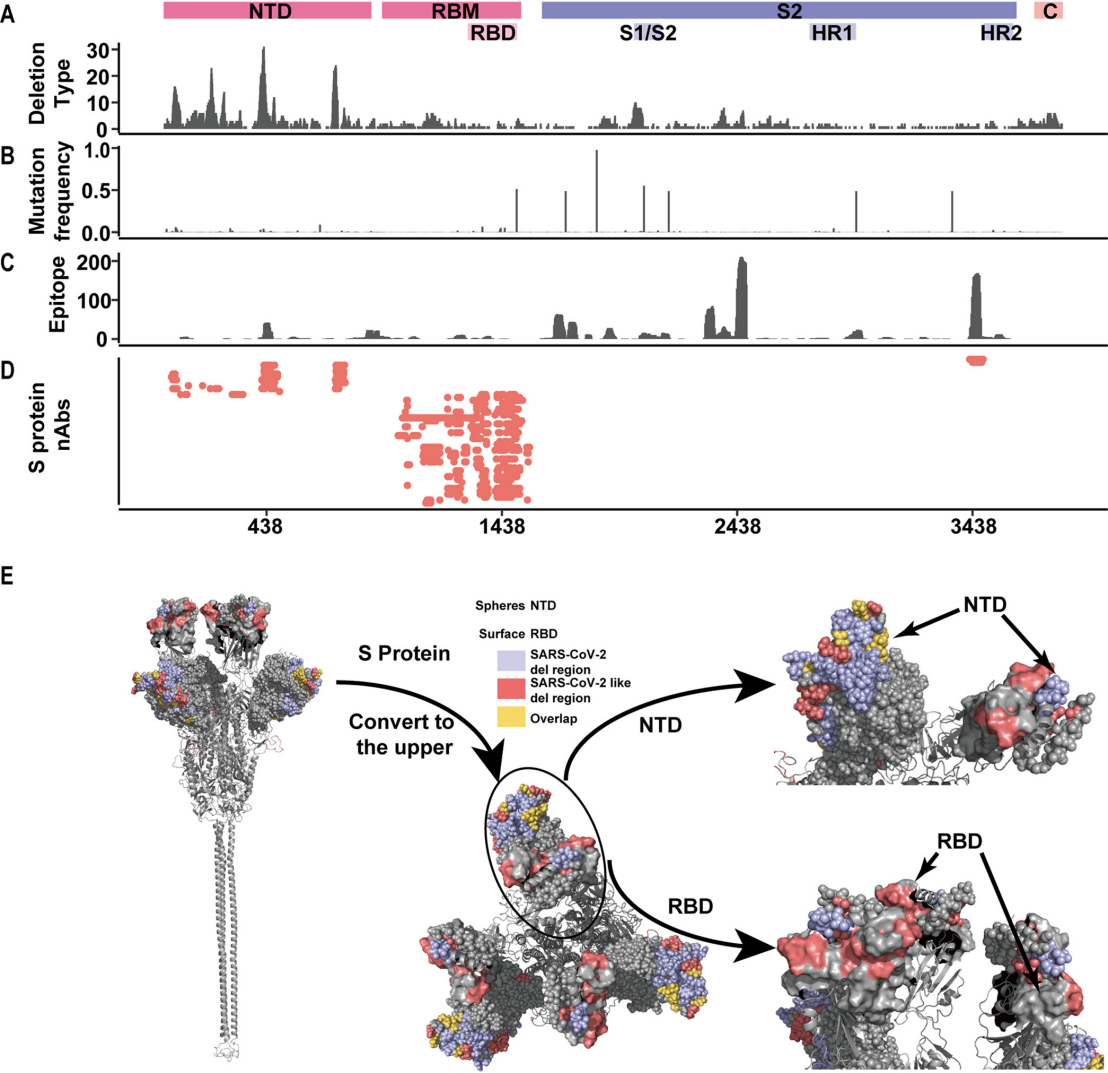
Relationships between deletions, mutations, and antibody binding regions in the S protein of SARS-CoV-2. (A–D) The distribution of deletions (A), mutations (B), IgA and IgG epitopes (C), and known neutralizing antibodies (D) on the SARS-CoV-2 S protein. (E) The RDRs are displayed on the 3-D model of the SARS-CoV-2 S protein. NTD and RBD are labeled as spheres and surfaces. The RDRs of SARS-CoV-2 (blue) and SARS-CoV-2-like viruses (red) are colored, and their overlapping area is in yellow.

To explore whether these deletions could influence viral antigenicity or change the binding regions for neutralizing antibodies, we first analyzed the relationship between deletions, mutations, antigenic sites, and the binding regions for neutralizing antibodies in the S protein (Fig. 4 and Fig. S3). We found that the high-prone mutation regions staggered to the RDRs. The NTD of the S protein was a high-risk region for deletions, while multiple high-frequency mutations were found in the S2 part of the S protein (Fig. 4A and B). In SARS-CoV-2, IgG and IgA epitopes mainly located in the S2 domain (Fig. 4C) (19). Previous studies had proved that most of the mutations cannot change the antigenic site of the virus (20). We further collected the currently reported neutralizing antibodies for the SARS-CoV-2 S protein. These antibodies could be divided into three types according to their binding sites (NTD, RBM, and HR2) (Table S5). Deletions in the NTD partially overlapped with the binding sites of these neutralizing antibodies (Fig. 4D). When we mapped RDRs and HDAs to the 3-D structure of the S protein, we found that, in SARS-CoV-2-like viruses, HDAs mainly aggregated in the RBD. In SARS-CoV-2, however, RDRs were mainly in the NTD. RDRs and HDAs partially overlapped in the NTD covering S Δ69-70. (Fig. 4E). The observed deletion site distribution was different in SARS-CoV-2 and SARS-CoV-2-like viruses. However, since all these strains belong to the same species, the observed variation may be due to the limited evolutionary time. Given a long period of evolution, deletions in SARS-CoV-2 and SARS-CoV-2-like viruses may tend to a similar pattern. All the results above indicated that some deletions in the S protein may contribute to the viral adaption, including the viral transmissibility and immune escape (20).

## DISCUSSION

Deletions were frequently and widely occurring in SARS-CoV-2, yet recent studies mainly focused on the S protein. Our analyses showed an overall distribution profile of deletions over the entire SARS-CoV-2 genome. We found that these deletions had a significant regional preference. We further extended this study to SARS-CoV-2-like and SARS-CoV-like viruses, finding that in these sequences deletion and insertion events also had a regional preference. These results implied that RDRs may have played a role in the evolution of SARS-CoV-2 and SARS-related coronaviruses.

Within all RDRs, four high-frequency deletions were detected majorly in the Alpha variant, which indicated the rapid increase of these deletions was because of the widespread outbreak of Alpha strain. Among these four deletions, S Δ144 was already proved to be involved in the viral escape from neutralizing antibodies (15). S Δ69-70 was involved in the increasement of cell entry efficiency (12). However, the function of middle-frequency deletions was still unclear. Furthermore, the cooperation of these deletions with some SNPs may play a certain role in the SARS-CoV-2 adaption and evolution. Therefore, further studies were urgently needed to understand their role in viral evolution and transmission.

The RNA-RNA interaction may trigger these deletions during viral replication. Lei et al identified the SARS-CoV-2 RNA genome structure by icSHAPE and found a large number of RNA-RNA interaction regions. Omer et al. found that SARS-CoV-2 had many short- or long-distance RNA-RNA interactions within cells (21). A study revealed the structural variants were enriched in the transcription regulatory site (TRS) of the SARS-CoV-2 genome (17). Here, we also found that a portion of identified RDRs was also located in front of the ORFs. More studies are required to reveal the reasons for the occurrence of deletions and their location preference.

The occurrence of deletions has been shown to lead to the immune escape of SARS-CoV-2 strains from neutralizing antibodies such as 4A8 (15). In this study, we found that the neutralizing antibody binding sites, which were mainly located at the NTD and RBD of SARS-CoV-2, overlapped with RDRs in the NTD domain. Furthermore, these RDRs and mutations in the S protein were present in a staggering arrangement. RDRs appeared mostly in the NTD of the S protein, while most of the high-frequency mutations presented in the S2. The complementary relationship between deletions and mutations indicated that SARS-CoV-2 evolved through using deletions to partly escape host immunity. The deletions with regional preference may work synergistically with other mutations to yield more comprehensive and rapid adaptability.

Since current SARS-CoV-2 vaccines were mainly developed against the S protein, these insertion and deletion speculations raise many questions. For instance, whether the development of vaccines can tolerate these SVs? Are the vaccines already on the market significantly weakened or partly weakened due to these deletions? Some studies have proved the role of the deletions in the S protein in viral adaption, especially in the changing NTD antigenicity from potently neutralizing convalescent plasma or specific neutralizing antibodies (20). The deletions on S Δ144/145 and S Δ243-244 were confirmed at the binding sites of a neutralizing antibody 4A8. These two deletions were proved to have the ability to abolish 4A8 binding (15). Therefore, these SVs (deletions and insertions) require careful monitoring and tracking in the future.

## MATERIALS AND METHODS

### Sequence source.

The aligned SARS-CoV-2 sequences were acquired on July 8, 2021, from the GISAID database (22). All sequences were collected before July 5, 2021. The sequences longer than 29000 nt have already been aligned using MAFFT in the GISAID database. After downloading these aligned sequences, quality control was operated according to the sequence quality standard following National Information Center, together with a host screening. Sequences owing more than 15 Ns or 50 merged bases were discarded and only those isolated from human samples were kept. There were finally 1,289,583 sequences used in deletion and insertion identification and further analysis. At the same time, we collected from GISAID the pedigree information, sampling time, and location information of these SARS-CoV-2 sequences.

### Insertion, deletion, and mutation identification.

Sequences related to SARS-CoV and SARS-CoV-2 were collected and treated by the tool 'Genome-to-Variants’ from the website of the China National Center for Bioinformation (https://ngdc.cncb.ac.cn/ncov/online/tool/variation) to obtain mutation information (23). The tool aligns each sequence to its reference sequences and then lists variations in VCF format. The reference sequences for SARS-CoV-like and SARS-CoV-2-like sequences were NC_004718.3 (NCBI Reference Sequence) and EPI_ISL_402124 (GISAID Reference Sequence), respectively. The insertion and deletion locations were extracted from the VCF file. Since this online tool is hard to treat a big dataset of SARS-CoV-2, we used an R script to treat the mega sequence data which was described in Fig. S4 in the supplemental material Fig. S4 The sequence named EPI_ISL_402124 was used as a reference sequence to extract the mutations and SVs information. We pulled out the reference sequence and compared it to the other sequences one by one. To avoid interference with sequencing quality, only the sites with a gap against normal bases (A, T, C, and G) will be treated as insertions or deletions. The R script is available at https://github.com/wuaipinglab/genome_treatment.

### Phylogenetic tree analysis.

The phylogenetic trees of SARS-CoV-like and SARS-CoV-2-like viruses were built by the ORF1b and constructed with the software ‘FastTree’ with version 2.1.9 (24) with the parameters “Fasttree-gtr-nt.” The SARS-CoV-2 phylogenetic tree was also constructed using FastTree software using their full-length sequences with the same parameters. The phylogenetic tree for each high-frequency deletion was shown on a background. The background sequence for viral evolution was composed of the latest sequence in each PANGO lineage. The PANGO lineage with deletions was highlighted in red.

### Recurrent deletion region identification.

To assemble a reasonable recurrent region, all the deletions that happened less than five times were removed. The remained deletions were joined by their location on the SARS-CoV-2 reference genome. The assembled area was defined as recurrent deletion regions (RDRs). For further analysis of the characteristic of these RDRs, the counts of the deletion types in each RDR were recorded. In SARS-CoV-like viruses, the insertion and deletion positions were uniformly corrected, based on the starting position of each protein against SARS-CoV-2.

### RDR visualization in protein structures.

The simulated S protein structure, which belongs to lineage A, was created by Zhang Yang lab’s website (http://zhanglab.ccmb.med.umich.edu/COVID-19/). The 3D structure visualization was done in PyMOL (25).

### Neutralizing antibody.

The SARS-CoV-2 antibody information was collected from the coronavirus antibody database CoV-AbDab (26). The antibodies owing the ability to neutralize the SARS-CoV-2 virus were selected. Their target sites on the virus were collected from their original research articles which were listed in Table S3 in the supplemental material. We used an R script to display these neutralizing antibodies with detailed binding positions on the S protein.
