# Supplementary material for: Conserved Pattern and Potential Role of Recurrent Deletions in SARS-CoV-2 Evolution
Source: Microbiol Spectr. 2022 Mar 7;10(2):e02191-21. doi: 10.1128/spectrum.02191-21 (PMC9045279; doi:10.1128/spectrum.02191-21)
Supplement: SUPPLEMENTAL FILE 6 — Supplemental material. Download SPECTRUM02191-21_Supp_6_seq11.pdf, PDF file, 4.2 MB [file spectrum02191-21_supp_6_seq11.pdf]

Supplemental Material

Conserved Pattern and Potential Role of Recurrent Deletions in SARS-CoV-2 Evolution

Shenghui Weng[1,2], Hangyu Zhou[1,2], Chengyang Ji[1,2], Liang Li[3], Na Han[1,2], Rong Yang[1,2], Jingzhe Shang[1,2]*, Aiping Wu[1,2]*

[1] Institute of Systems Medicine, Chinese Academy of Medical Sciences & Peking Union Medical College, Beijing, 100005, China. [2] Suzhou Institute of Systems Medicine, Suzhou, 215123, China. [3] Linyi People's Hospital, Shandong, 276000, China.

* To whom correspondence should be addressed. Email: Aiping Wu (wap@ism.cams.cn) and Jingzhe Shang (sjz@ism.cams.cn).

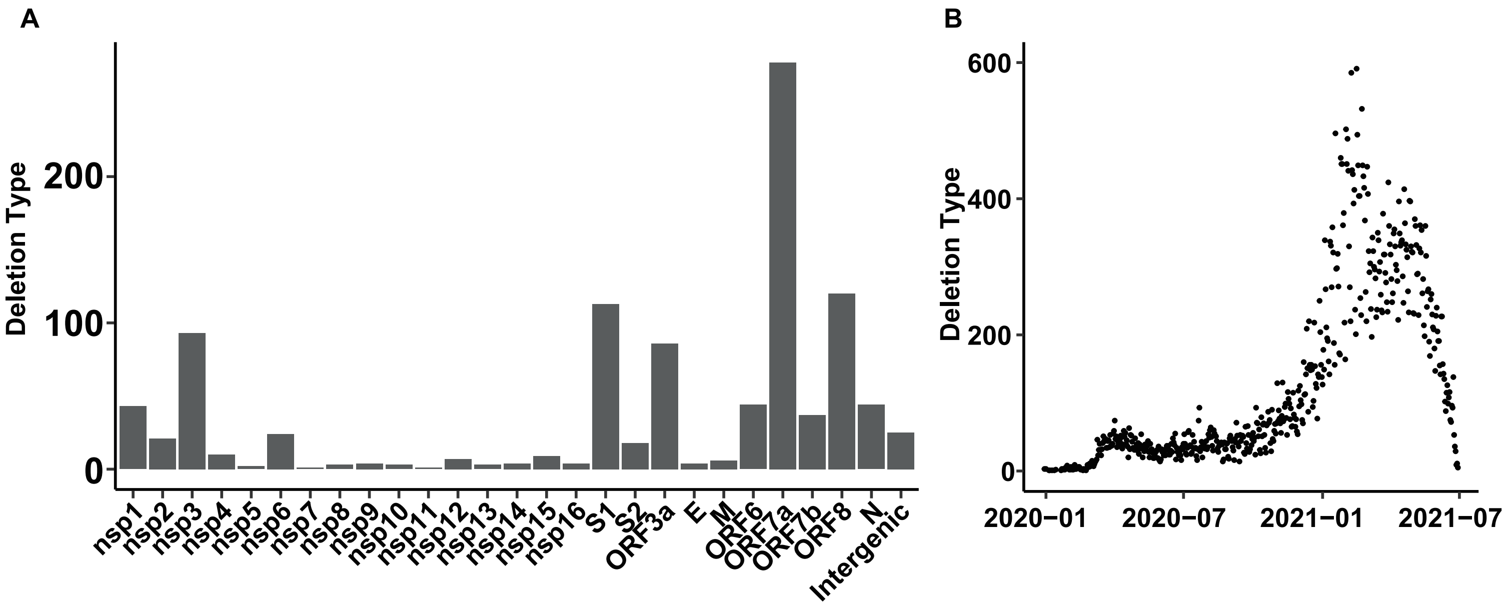

Supplemental Figure 1. Deletion types of SARS-CoV-2. (A) The number of deletion types in each protein in SARS-CoV-2 is shown. (B) The number of deletion types changes over time in SARS-CoV-2.

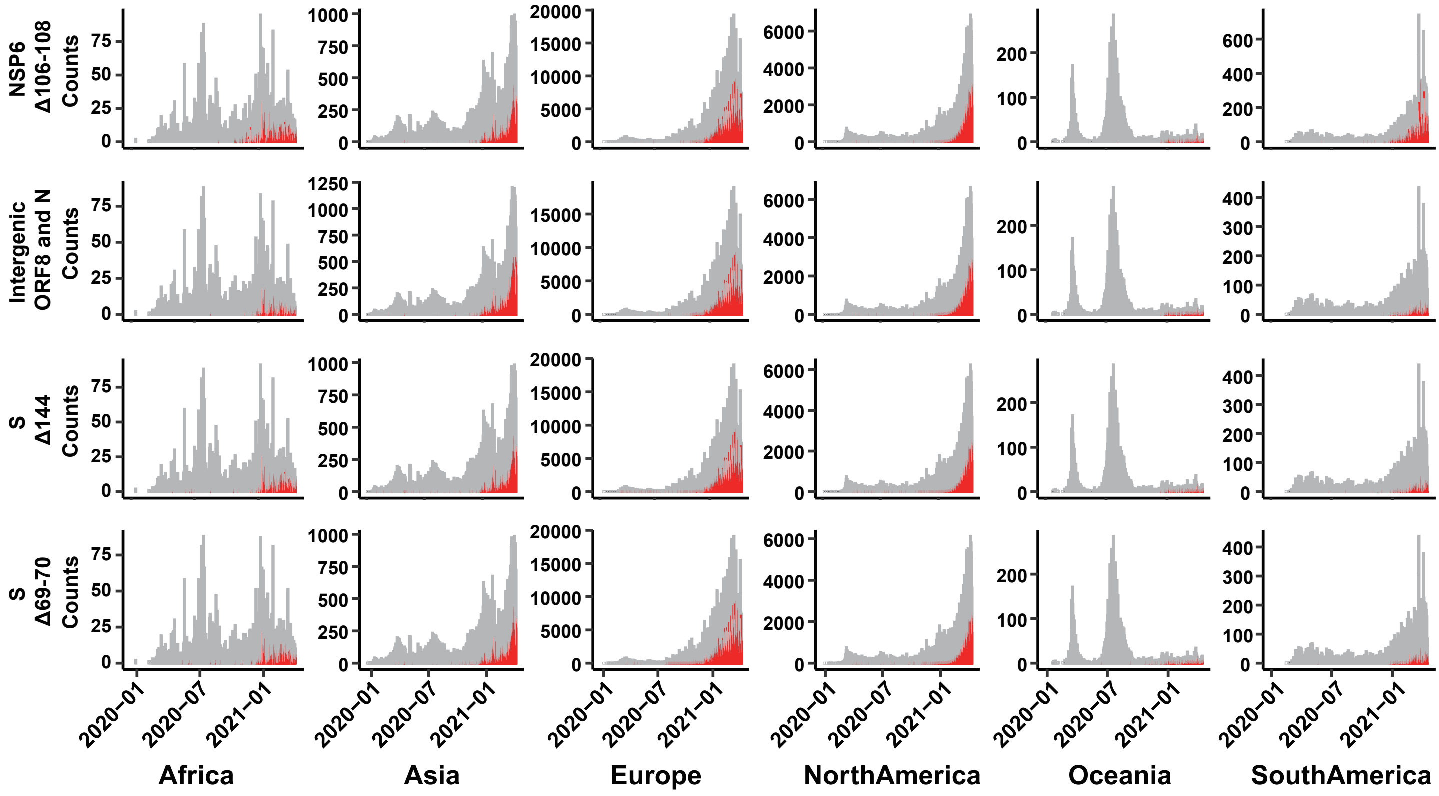

Supplemental Figure 2. The spatiotemporal distribution of high-frequency deletion in the SARS-CoV-2 genome. The number of SARS-CoV-2 variants with four high-frequency deletions changed over time in six continents. Sequences with specific deletions were marked in red, and other sequences were in grey.

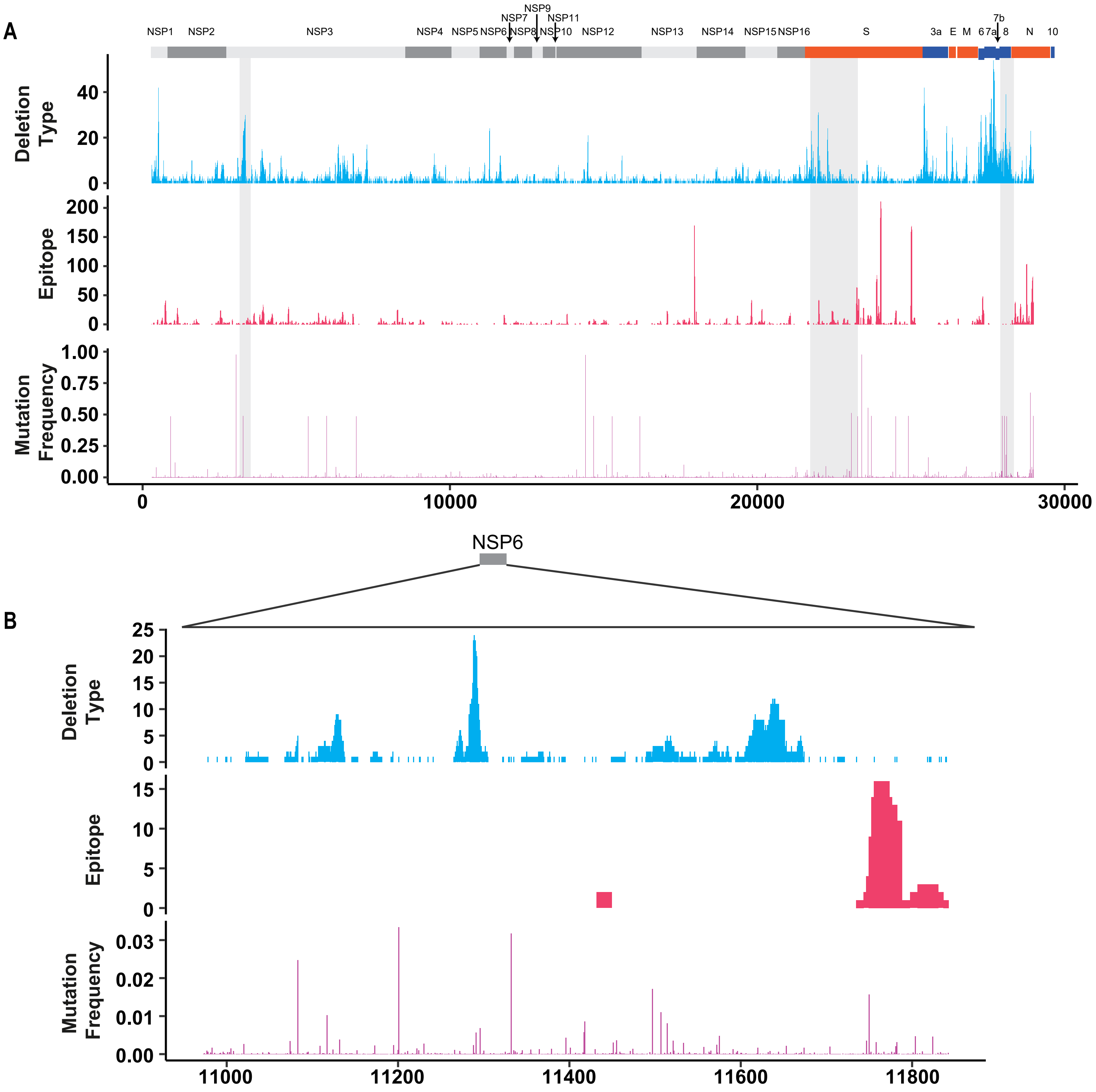

Supplemental Figure 3. The RDRs on the SARS-CoV-2 genome and their relationship with antigenic sites and mutations. The distribution of deletions over the entire genome (A) and nsp6 (B) of SARS-CoV-2 is shown. Antigenic sites and mutations are also shown.

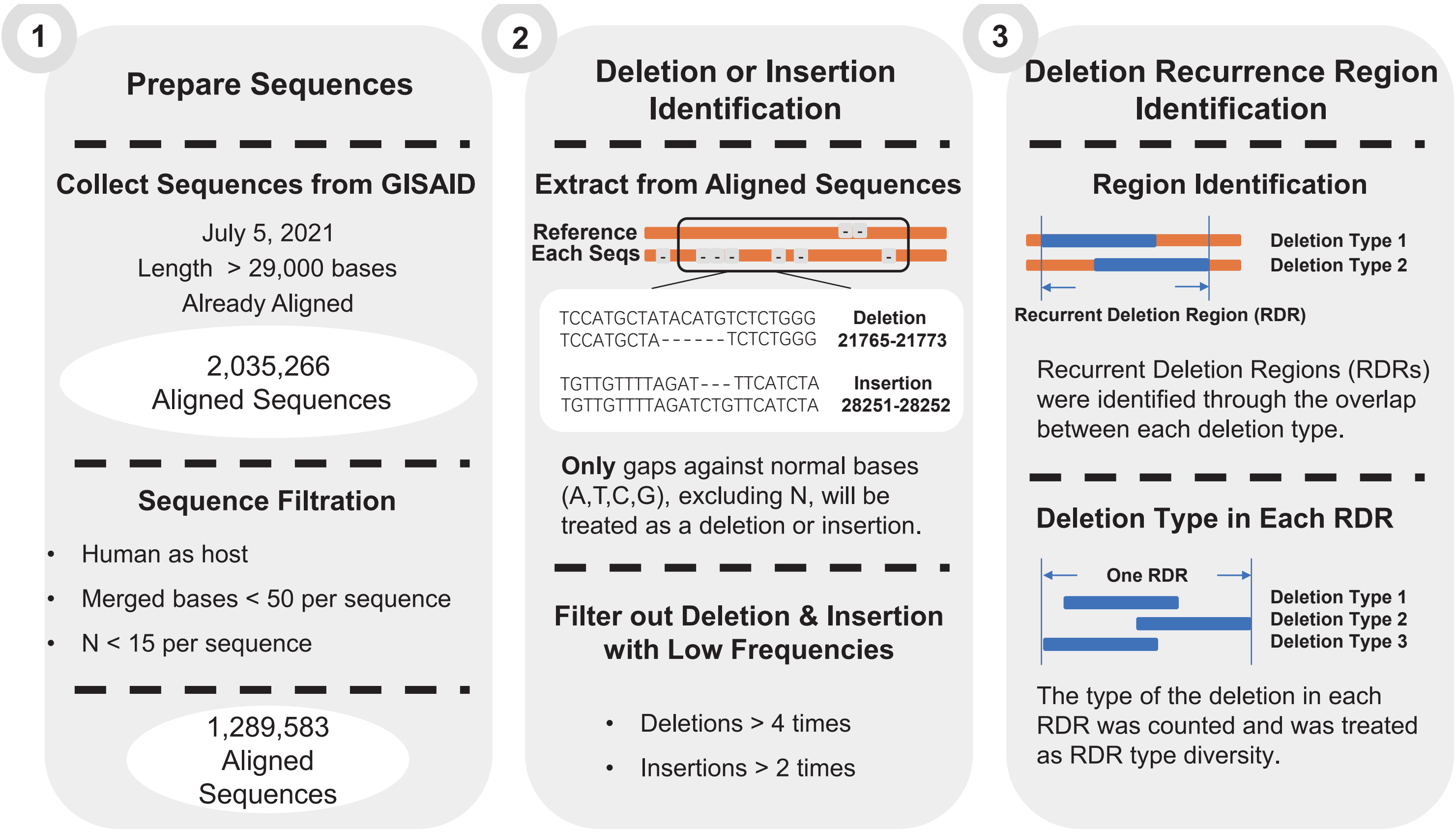

Supplemental Figure 4. Methodology of RDR identification. The workflow of (1) sequence preparation, (2) deletion and insertion identification, and (3) deletion recurrence region identification is shown in detail. The SARS-CoV-2 sequences were downloaded from the GISAID database on July 8, 2021. All the sequences were collected before July 5, 2021. GISAID only provided sequences longer than 29000 bases in their aligned sequence file. These sequences were aligned by mafft, and further detail can be found on the GISAID website. From the original 2,035,266 aligned SARS-CoV-2 sequences, 1, 289, 583 sequences were filtered out. Only the sequences isolated from humans, whose merged bases are less than 50 and N are less than 15, will be used. In these sequences, deletion and insertion from each sequence were identified by comparing these sequences to a reference sequence one by one. EPI_ISL_402124 was used as the reference. Only a gap mapping to normal bases (A, T, C, G) was screened out as a deletion or insertion. In further analysis, the deletions occurred more than 4 times, and the insertions more than 2 times were operated. The remained deletions were used to form the recurrent deletion regions. When the deletion type overlapped, a region consisting of these deletions were treated as a recurrent deletion region. At the same time, the number of deletion types in each region was recorded.
